# SARS-CoV-2 modulates inflammatory responses of alveolar epithelial type II cells *via* PI3K/AKT pathway

**DOI:** 10.3389/fimmu.2022.1020624

**Published:** 2022-10-31

**Authors:** Ahmed A. Al-Qahtani, Ioanna Pantazi, Fatimah S. Alhamlan, Hani Alothaid, Sabine Matou-Nasri, George Sourvinos, Eleni Vergadi, Christos Tsatsanis

**Affiliations:** ^1^ Department of Infection and Immunity, Research Center, King Faisal Specialist Hospital and Research Center, Riyadh, Saudi Arabia; ^2^ Department of Microbiology and Immunology, College of Medicine, Alfaisal University, Riyadh, Saudi Arabia; ^3^ Laboratory of Clinical Chemistry, Medical School, University of Crete, Heraklion, Greece; ^4^ Department of Pediatrics, Medical School, University of Crete, Heraklion, Greece; ^5^ Department of Basic Sciences, Faculty of Applied Medical Sciences, Al-Baha University, Al-Baha, Saudi Arabia; ^6^ Cell and Gene Therapy Group, Medical Genomics Research Department, King Abdullah International Medical Research Center, King Saud bin Abdulaziz University for Health Sciences, Ministry of National Guard Health Affairs, Riyadh, Saudi Arabia; ^7^ Laboratory of Virology, Medical School, University of Crete, Heraklion, Greece; ^8^ Institute of Molecular Biology and Biotechnology, Foundation for Research and Technology (FORTH), Heraklion, Greece

**Keywords:** alveolar epithelial cells, COVID-19, SARS-CoV-2, AKT, inflammation

## Abstract

**Background:**

SARS-CoV-2 infects through the respiratory route and triggers inflammatory response by affecting multiple cell types including type II alveolar epithelial cells. SARS-CoV-2 triggers signals *via* its Spike (S) protein, which have been shown to participate in the pathogenesis of COVID19.

**Aim:**

Aim of the present study was to investigate the effect of SARS-CoV2 on type II alveolar epithelial cells, focusing on signals initiated by its S protein and their impact on the expression of inflammatory mediators.

**Results:**

For this purpose A549 alveolar type II epithelial cells were exposed to SARS CoV2 S recombinant protein and the expression of inflammatory mediators was measured. The results showed that SARS-CoV-2 S protein decreased the expression and secretion of IL8, IL6 and TNFα, 6 hours following stimulation, while it had no effect on IFNα, CXCL5 and PAI-1 expression. We further examined whether SARS-CoV-2 S protein, when combined with TLR2 signals, which are also triggered by SARS-CoV2 and its envelope protein, exerts a different effect in type II alveolar epithelial cells. Simultaneous treatment of A549 cells with SARS-CoV-2 S protein and the TLR2 ligand PAM3csk4 decreased secretion of IL8, IL6 and TNFα, while it significantly increased IFNα, CXCL5 and PAI-1 mRNA expression. To investigate the molecular pathway through which SARS-CoV-2 S protein exerted this immunomodulatory action in alveolar epithelial cells, we measured the induction of MAPK/ERK and PI3K/AKT pathways and found that SARS-CoV-2 S protein induced the activation of the serine threonine kinase AKT. Treatment with the Akt inhibitor MK-2206, abolished the inhibitory effect of SARS-CoV-2 S protein on IL8, IL6 and TNFα expression, suggesting that SARS-CoV-2 S protein mediated its action *via* AKT kinases.

**Conclusion:**

The findings of our study, showed that SARS-CoV-2 S protein suppressed inflammatory responses in alveolar epithelial type II cells at early stages of infection through activation of the PI3K/AKT pathway. Thus, our results suggest that at early stages SARS-CoV-2 S protein signals inhibit immune responses to the virus allowing it to propagate the infection while in combination with TLR2 signals enhances PAI-1 expression, potentially affecting the local coagulation cascade.

## Introduction

During the last two decades, public health faced several outbreaks of severe respiratory diseases caused by coronaviruses, such as severe acute respiratory syndrome coronavirus (SARS-CoV) and Middle East respiratory syndrome coronavirus (MERS-CoV), which were soon held under control. However, the outbreak of the newer SARS-CoV-2 coronavirus at the end of 2019 in Wuhan City, which causes COVID-19 disease, evolved rapidly to a global pandemic with unprecedented health and economic repercussions. A major complication characterizing COVID-19 pathogenesis, is the presence of microvascular and macrovascular thrombosis which are associated with a high risk of mortality (1, 2). For example, the formation of thrombi in pulmonary vessels can lead to pulmonary embolism and acute respiratory failure observed in patients with COVID-19 (3). Impaired fibrinolysis also raises the thrombotic risk observed in COVID-19 patients. Fibrinolytic activity is regulated by plasminogen activators (tPA, uPA) and plasminogen activator inhibitor-1 (PAI-1), which serve as useful biomarkers of the disease (4).

SARS-CoV-2 cell entry is achieved through binding of its surface spike protein to its main cellular receptor angiotensin converting enzyme 2 (ACE2) (5–7). The spike protein is a homotrimeric class I fusion protein consisting of a receptor-binding subunit S1 and a membrane-fusion subunit S2 (8–10). When SARS-CoV-2 binds to ACE2 with S1 subunit, the spike protein undergoes protease cleavage at the S1/S2 cleavage site by the transmembrane Serine Protease 2 (TMPRSS2), allowing fusion of the viral membrane with the host-cell membrane by the S2 subunit, and the subsequent viral endocytosis (5, 11, 12). In addition to its direct binding to ACE2, the S protein is a potent viral PAMP that is sensed by other cell receptors, such as TLR2 in lung epithelial cells (13). Subsequently, TLR2 forms heterodimers with TLR1 or TLR6, creating a complex containing MyD88 with IRAK kinase family members, leading to activation of NF-κB and MAPK signaling, and ultimately to the production of inflammatory cytokines and chemokines (14). The expression of TLR2 is also increased following SARS-CoV-2 infection and it is positively associated with the severity of COVID-19 (15). In addition to the S protein, TLR2 senses SARS-CoV-2 envelope protein, inducing the production of proinflammatory cytokines independent of viral entry (15).

The initial site of infection and viral replication is the sinonasal airway epithelium, consisting of ciliated and mucus secretory cells (16). As the disease spreads down to the alveolar compartment, the primary cell being infected by SARS-COV-2 is the alveolar type II cell, which is also the main cell type that expresses ACE2 and TMPRSS2 in the lung (17, 18). After SARS-CoV-2 infection, alveolar type II cells release the virus that infects adjacent type II cells, and also secrete interferons and inflammatory cytokines and chemokines to initiate the innate immune response. The inflammatory response includes mobilization of immune cells and tissue damage. The ultimate consequence is diffuse alveolar injury with loss of functional surfactant, damage of type I cells and endothelial cells, alveolar flooding and influx of inflammatory cells (19, 20). Disease severity is associated to a highly dysregulated innate immune response, characterized by this excessive inflammatory response, as well as a relatively delayed interferon (IFN) response against the virus, facilitating robust viral replication and inflammatory damage to tissues (21). In contrast, multiple studies show that critically ill COVID-19 patients are characterized by lymphopenia with loss of CD4+ T, CD8+ T, NK cells and B cells, as well as a decreased number of immune cells producing IFNγ and TNFα (22–24). There are several possible explanations for this, including pulmonary recruitment of lymphocytes from the blood, direct virus killing of lymphocytes, T-cell apoptosis and exhaustion (25). Furthermore, other studies have also shown an increased release of anti-inflammatory cytokines and mediators, such as IL10 and cytokine growth and differentiation factor 15 (GDF-15) in COVID-19 patients, possibly as a mechanism to downregulate excessive inflammatory responses and restore the balance between pro and anti-inflammatory responses (26–28). Therefore, COVID-19 infection cannot be characterized by a classical cytokine storm syndrome, but rather as a significant inflammatory dysregulation with alternating hyperinflammation and immunosuppression states during the infection. Understanding which immune state predominates at each stage of SARS-CoV-2 infection, as well as the molecular pathways that regulate them, is very crucial in order to enhance our knowledge of disease pathogenesis and develop new therapeutic strategies.

During SARS-CoV-2 infection, several signaling pathways are activated by the interaction of spike protein with its ACE2 receptor. Specifically, it has been shown that SARS-CoV-2 infection can cause multiple intracellular phosphorylation events in the mTOR, ERK and JAK1 pathways (29–31). The PI3K/Akt/mTOR pathway is an important cell signaling pathway that regulates various cell functions and its upregulation has been observed in diseases caused by viruses (32). Following the activation of related receptors by viruses, PI3K generates PIP3, resulting in the activation of PDK1 which further activates protein kinase B (PKB/Akt). The phosphorylated Akt mediates the phosphorylation of mTOR, promoting nuclear translocation of NF-κB, which regulates proinflammatory gene expression. The role of the PI3K/Akt pathway in cytokine production is cell-type specific and depends on the stimulus applied. Therefore, PI3K/Akt might exert proinflammatory or anti-inflammatory properties according to the situation. Several studies have already demonstrated the implication of this pathway in COVID-19 pathogenesis. For example, activation of the PI3K/Akt signaling pathway can be induced by CD147 and furin (involved in SARS-CoV-2 cell entry), while the clathrin-mediated SARS-CoV-2 endocytosis is also regulated by the PI3K/Akt pathway (33). Another proteotranscriptomics study, showed increased levels of phosphorylation of Akt, mTOR and other downstream effectors at 24h post-infection, indicating activation of Akt-mTOR pathway at an early stage of the infection (30).

In the present study, we investigated the impact of SARS-CoV-2 S protein in the production of inflammatory mediators alone or in combination with TLR2 signals from alveolar epithelial type II cell. We further, investigated the possible molecular pathways (MAPK/ERK, PI3K/Akt) implicated in this process, in order to determine how SARS-CoV-2 influences its main entry site at an early stage of the infection.

## Materials and methods

### Cell culture

The A549 cell line (ATCC: CCL-185) was used in this study as a model of type II alveolar epithelial cells. Cells were cultured in Dulbecco Modified Eagle Medium (DMEM), low glucose (1g/L) (Thermo Fisher Scientific, Waltham, USA) supplemented with 10% fetal bovine serum (FBS) and antibiotics (10,000 U/ml penicillin and 10 mg/ml Streptomycin). Cells were seeded in 24-well plates at a final density of 4×10^5^ cells/ml and were stimulated with different concentrations of SARS-CoV-2 Spike-Membrane Recombinant Fusion Protein (10, 20, 50, 100 ng/ml; TP701119, OriGene, Rockville, USA) in the presence or absence of the TLR ligand PAM3csk4 (1μg/ml; Tocris, Bristol, UK) for 6 and 12 hours. In another set of experiments, A549 cells were pre-treated with the selective Akt 1/2/3 inhibitor MK-2206 2HCl (5μM; cat# S1078, SelleckChem, Berlin, Germany) for 24 hours and then stimulated with 50ng/ml SARS-CoV-2 Spike-Membrane Recombinant Fusion Protein for 18 hours to collect cell culture supernatants.

### Enzyme-linked immunosorbent assay (ELISA)

A549 cells were stimulated with SARS-CoV-2 Spike-Membrane Recombinant Fusion Protein for 12 hours in the presence or absence of TLR2 ligand, and cell culture supernatants were collected for cytokine quantification. Cytokine production of IL6, IL8 and TNFα was determined using the Elisa Max™ Delux Set (BioLegend, SanDiego USA) as indicated by the manufacturer.

### Real-time PCR

For the mRNA level detection of IL6, IL8, TNFα, CXCL5, PAI-1 and IFNα, total RNA was extracted from A549 cells using TRI Reagent (Sigma-Aldrich, St Louis, USA). Eight hundred nanogram of total RNA were used for cDNA synthesis (TAKARA, Primescript RT Reagent kit, Tokyo, Japan). Amplification was performed using KAPA SyBr^®^ Fast Universal qPCR kit (Kapa Biosystems, Cape Town, South Africa). Denaturation was carried out at 95°C for 10 seconds, annealing and extension at 60°C for 30 seconds for 40 cycles in a StepOnePlus™ Real-Time PCR System (Applied Biosystems, Foster City, CA, USA). Data analysis was accomplished using the ΔΔCT method and GAPDH was used as the housekeeping gene. The primer sequences used in this study, were the following: IL6: forward 5’ GTCAGGGGTGGTTATTGCAT 3’ and reverse 5’ AGTGAGGAACAAGCCAGAGC 3’; IL8: forward 5’ TGTGAAGGTGCAGTTTTGCC 3’ and reverse 5’ CACCCAGTTTTCCTTGGGGT 3’; TNFα: forward 5’ GCCCAGGCAGTCAGATCAT 3’ and reverse 5’ TATCTCTCAGCTCCACGCCA 3’; CXCL5: forward 5’ ACAGACCACGCAAGGAGTTC 3’ and reverse 5’ TCTTCAGGGAGGCTACCACT 3’; PAI-1: forward 5’ TCACGAGTCTTTCAGACCAAG 3’ and reverse 5’ CCGGACCACAAAGAGGAAG 3’; IFNα: forward 5’ GGAGGAGAGGGTGGGAGAAA 3’ and reverse 5’ GACAACCTCCCAGGCACAAG 3’; GAPDH: forward 5’ GGAAGGTGAAGGTCGGAGTCA 3’ and reverse 5’ GTCATTGATGGCAACAATATCCACT 3’.

### Western blot

For Western blot, cell lysates were harvested with RIPA lysis buffer and protein concentration was determined using the Pierce BCA Protein Assay. Protein lysates were resuspended in SDS-containing loading dye, were separated on 10% polyacrylamide gel, and then transferred to nitrocellulose membrane. Briefly, after blocking with 5% Bovine Serum Albumin (BSA) containing 0.1% Tween 20 for an hour at room temperature, the membranes were incubated overnight at 4^0^C with primary antibodies (1:1000), washed with PBST and then incubated with peroxidase-conjugated secondary antibodies (1:5000) for 1 hour at room temperature. Membranes were exposed to trans-UV light in a ChemiDoc XRS+ (BioRad Laborato-ries, Inc, Hercules, CA, US) and signals were digitalized and analyzed by densitometry with the embedded software (Image Lab Software, v.6.1). Band intensities of phosphorylated proteins were normalized with total protein intensities as well as with the loading control beta-actin. The antibodies used in this study were the following: p-Akt (cat#9271, Cell Signaling, Massachusetts, USA), Akt (Cell Signaling, #9272, Massachusetts, USA), p-Erk 1/2 (Cell Signaling, #9101, Massachusetts, USA), Erk 1/2 (Cell Signaling, #9102, Massachusetts, USA) and β-actin (Cell Signaling, #3700, Massachusetts, USA).

### Statistical analysis

Comparison among groups was performed using t-test for parametric data, or Mann—Whitney and the Kruskal—Wallis test with Dunn’s multiple comparison post-test for non-parametric data. Data were depicted in box-and-whiskers or bars and plotted as median with range or mean ± S.D. The GraphPad InStat Software (GraphPad v6.0, San Diego, CA, USA) was used for analysis. P value < 0.05 was considered statistically significant. Results are representative of at least three independent experiments.

## Results

### SARS-CoV-2 S protein suppresses pro-inflammatory responses in A549 epithelial cells

**Figure 1:**
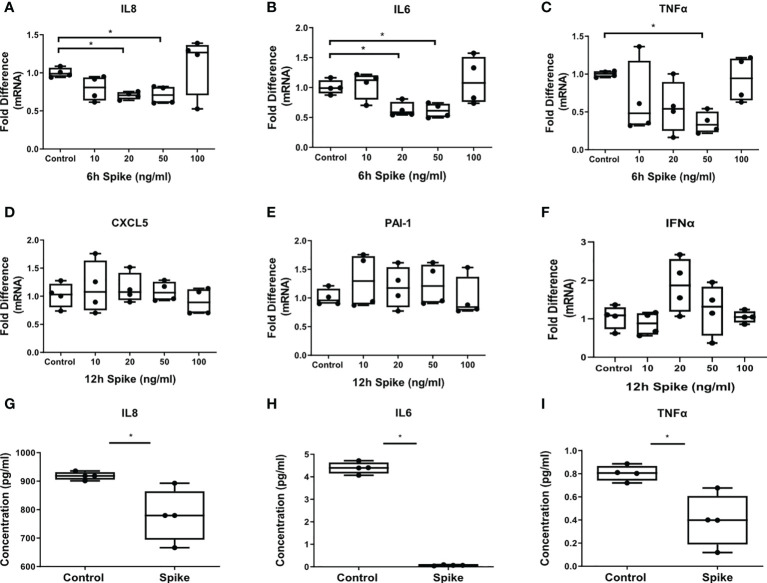
SARS-CoV-2 S protein suppressed the expression and production of pro-inflammatory cytokines in A549 cells. A549 cells were treated with different concentrations of SARS-CoV-2 Spike-Membrane recombinant fusion protein (10, 20, 50, 100 ng/ml) for 6 or 12 hours. **(A–C)** mRNA expression of IL6, IL8 and TNFα decreased upon SARS-CoV2 Spike stimulation for 6 hours compared to untreated control cells. **(D–F)** No effect was observed for the expression of CXCL5, PAI-1 and IFNα at 12 hours following stimulation. **(G–I)** Secretion of IL8, IL6 and TNFα decreased upon SARS-CoV2 Spike stimulation (50ng/ml) for 12 hours. Data are illustrated in box-and-whiskers and plotted as median with range (n=4 biological replicates per group). Statistical analysis was performed with Kruskal - Wallis test **(A–F)** and Mann – Whitney U test **(G–I)**. Results are representative of three independent experiments. *p < 0.05.

The inflammatory responses of alveolar epithelial cells driven by the SARS-CoV-2 -Spike/ACE2 interaction were determined by stimulation of A549 cells with different concentrations of SARS-CoV-2 Spike-Membrane recombinant fusion protein and measuring the induction of the inflammatory mediators IL6, TNFα, IL8 at 6 hours following stimulation, since they are induced early in inflammatory responses, and IFNα, CXCL5 and PAI-1 at 12 hours following stimulation, since they are induced at later time points. We utilized a commercially available SARS-CoV-2 S protein raised in HEK293 cells, therefore no bacteria were involved, to avoid potential endotoxin contamination. The results showed that SARS-CoV-2 S protein suppressed the expression and production of IL8, IL6 and TNFα (**Figures 1A–C**, **G–I**). At higher doses of the recombinant protein the suppressive effect was abrogated, possibly due to binding of the protein to additional receptors with lower affinity, such as TLRs, through which the effect could be stimulatory. Expression of CXCL5, PAI-1 and IFNα was measured at 6 and 12 hours following stimulation with SARS-CoV-2 S protein but no effect was observed (**Figures 1D–F**). The results showed that SARS-CoV-2 S protein suppressed the release of pro-inflammatory cytokines at early stages of contact with alveolar epithelial cells.

### SARS-CoV-2 S protein modulated TLR2 responses in A549 epithelial cells

**Figure 2:**
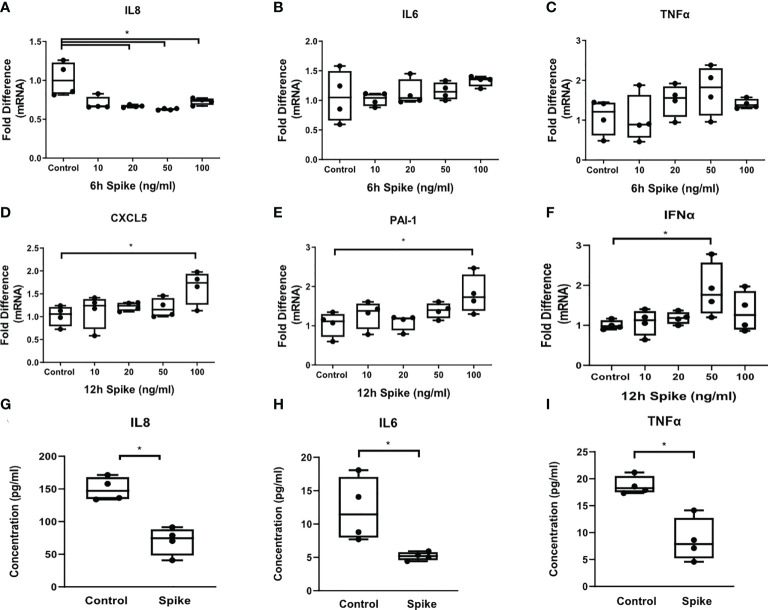
SARS-CoV-2 S protein differentially modulated TLR responses in A549 cells. A549 cells were treated with different concentrations of SARS-CoV-2 Spike-Membrane recombinant fusion protein (10, 20, 50, 100 ng/ml) and PAM3csk4 (1μg/ml) for 6 or 12 hours. **(A)** Decreased mRNA expression of IL8 was observed upon co-stimulation with SARS CoV2 S protein and PAM3csk4, compared to cells treated with PAM3csk4-alone (control). **(B, C)** No effect was observed for the expression of IL6 and TNFα. **(D–F)** Increased expression of CXCL5, PAI-1 and IFNα at 12 hours post-stimulation. **(G–I)** Secretion of IL8, IL6 and TNFα decreased in the presence of SARS-CoV-2 S (50ng/ml) after 12 hours. Data are illustrated in box-and-whiskers and plotted as median with range (n=4 biological replicates per group). Statistical analysis was performed with Kruskal - Wallis test **(A–F)** and Mann – Whitney U test **(G–I)**. Results are representative of three independent experiments. *p < 0.05.

To determine the effect of SARS-CoV-2 S protein on alveolar epithelial cells when co-stimulated with TLR2, a signal that can be initiated by SARS CoV2 envelope protein or by bacterial lipoproteins, we exposed A549 cells to the TLR2 ligand PAM3csk4 in the presence of SARS-CoV-2 Spike for 6 and 12 hours. PAM3csk4-stimulated A549 cells demonstrated reduced expression and secretion of IL8 (**Figures 2A, G**), as well as reduced secretion of IL6 and TNFα (**Figures 2H, I**), following stimulation with SARS-CoV-2 S protein. At the mRNA level, expression of IL6 and TNFα was not affected (**Figures 2B, C**), suggesting that regulation of these proteins by SARS-CoV-2 S protein may occur at the post-transcriptional level. On the contrary, PAM3csk4-induced expression of inflammatory mediators CXCL5, PAI-1 and IFNα increased at 12 hours post-stimulation with SARS-CoV-2 S protein (**Figures 2D–F**). Expression of CXCL5, PAI-1 and IFNα was not affected at 6 hours following stimulation (data not shown). The results showed that SARS-CoV-2 S protein suppressed the induction of pro-inflammatory cytokines in TLR2-stimulated A549 cells at early timepoints, while combination of SARS-CoV-2 S protein and TLR2 induced expression of the anti-viral responses through IFNα, expression of the chemokine CXCL5 potentially leading to recruitment of inflammatory cells.

### SARS-CoV-2 S exerted its immunosuppressive action through the PI3K/Akt pathway in A549 cells

**Figure 3:**
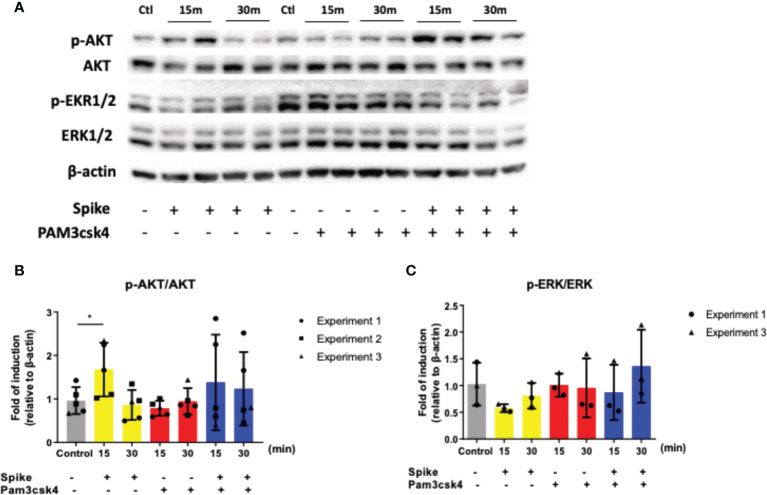
SARS-CoV-2 S protein triggered the induction of the PI3K/Akt pathway. A549 cells were treated with 50ng/ml SARS-CoV-2 S protein or PAM3csk4 or SARS-CoV-2 S and PAM3csk4 for 15 and 30 minutes. SARS-CoV-2 S protein slightly decreased the phosphorylation of ERK1/2 **(A, C)**, while significantly increased the phosphorylation of AKT **(A, B)**. Representative western blot **(A)** is presented and densitometry analysis from three **(B)** or two **(C)** independent experiments. Densitometry analysis is illustrated in bar graphs and plotted as mean ± S.D. Individual points indicate the biological replicates from all experiments (experiments 1 and 2 include two biological replicates for each condition and experiment 3 includes one biological replicate per condition). Statistical analysis was performed with t test **(B)**, but it was omitted for ERK due to the small sample size **(C)**. *p < 0.05.

To determine which signaling pathway is involved in the early SARS-CoV-2 S immunosuppressive action, we stimulated A549 cells with SARS-CoV-2 S protein, TLR2 ligand PAM3csk4 and the additive effect of both SARS-CoV-2 S protein and PAM3csk4 for 15 and 30 minutes for protein collection and quantification of specific signaling targets in western blot. The signaling pathways investigated were the PI3K/Akt and MAPK/ERK pathway, which were previously associated with COVID19 pathogenesis and progression. While MAPK/ERK is a well characterized pro-inflammatory pathway (34), Akt signals initiate both anti-inflammatory and pro-inflammatory effects (35). The results shown in **Figure 3**, revealed that SARS-CoV-2 S slightly decreased the phosphorylation and activation of ERK1/2, while it increased the phosphorylation and activation of AKT. The addition of TLR2 stimuli along with SARS-CoV-2 S amplified this effect considerably.

**Figure 4:**
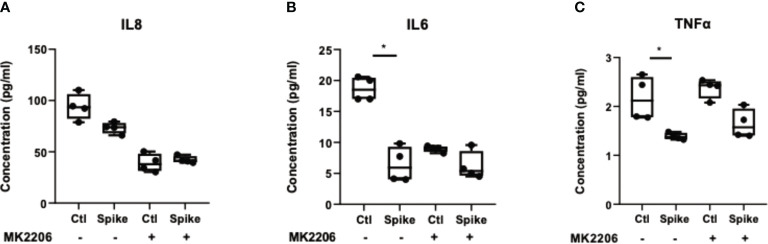
Inhibition of AKT abrogated the immunosuppressive action of SARS-CoV-2 S protein. A549 cells were treated with the pan-AKT inhibitor MK2206 (5μM) for 24 hours and then stimulated with SARS-CoV-2 S (50ng/ml) for 12 hours. Secretion of IL8 **(A)**, IL6 **(B)** and TNFα **(C)** decreased in the presence of SARS-CoV-2 S, an effect which was less evident in MK2206-treated cells. Data are illustrated in bars and plotted as median with range (n=4 biological replicates per group). Statistical analysis was performed with Kruskal - Wallis test. *p < 0.05.

To confirm that suppression of pro-inflammatory cytokines by SARS-CoV-2 S protein was mediated through the PI3K/Akt pathway, we treated A549 cells with the selective AKT inhibitor MK2206 for 24 hours and subsequently stimulated cells with SARS-CoV-2 S protein. Inhibition of Akt with MK2206 was confirmed by western blot (**Supplementary Figure 1**). In the presence of SARS-CoV-2 S protein, A549 cells secreted reduced IL8, IL6 and TNFα, an effect which was abrogated in MK2206-treated cells (**Figure 4**). This result confirmed that the initial immunosuppression observed in the presence of SARS-CoV-2 S protein was mediated through the PI3K/Akt pathway.

## Discussion

COVID-19 pandemic continues to pose a major challenge for public health and economy. Severe COVID-19 cases are associated with development of lung injury, vascular damage and ARDS, which is the main cause of high mortality rates (36). SARS-CoV-2 initial sites of infection include the upper and lower respiratory tracts. At the lower respiratory tract, SARS-CoV-2 infects cells of the gas exchange portion of the lung and in particular the alveolar epithelial type II cells. Pathological alterations at this region, include alveolar damage, pneumocyte desquamation and hyaline membrane formation, as well as significant accumulation of monocytes/macrophages, responsible for the robust inflammatory cytokine response observed (37, 38). However, recent studies have shown a dampened innate immune response after SARS-CoV-2 infection of nasal and bronchial epithelial cells (39, 40), as well as an early immunosuppression stage preceding the later hyperinflammation stage that leads to cytokine storm and ARDS (41). Therefore, developing new personalized therapeutic interventions requires a better understanding of the differential immune responses according to the cell type infected, as well as the COVID-19 patient’s immune status and how it evolves during the course of the disease.

In the present study, we used A549 cells stimulated with SARS-CoV-2 S protein as a model to investigate the immunomodulatory action of SARS-CoV-2 in alveolar epithelial type II cells. Our findings demonstrated decreased expression and secretion of IL8, IL6 and TNFα, while CXCL5, PAI-1 and IFNα remained unaffected even 12 hours post-infection. This finding implies that alveolar epithelial type II cells may enter an immunosuppressive state at early stages of the infection. Previous studies have already demonstrated reduced expression of pro-inflammatory cytokines in the myeloid cells from patients with COVID-19 (42), as well as reduced production of chemokines (41). A possible explanation for the early decreased inflammatory responses of the airway epithelium could be the result of immune evasion mechanisms employed by the virus to survive and replicate inside the alveolar epithelial cells avoiding the detection by other immune cells (43). Another potential explanation could be the occurrence of increased cell death and the disruption of tight junction complexes between adjacent epithelial cells observed during SARS-CoV-2 infection (41), leading to the upregulation of homeostatic mechanisms to resolve this situation rather than inflammatory responses. Co-stimulation of SARS-CoV-2 S protein with the TLR2 ligand PAM3csk4, increased significantly the expression of the inflammatory mediators CXCL5, PAI-1 and IFNα at 12 hours following infection, but could not change the decreased production of the pro-inflammatory cytokines IL8, IL6 and TNFα. While it is known that TLR2 signaling induces inflammatory responses via the NF-kB pathway during SARS-CoV-2 infection (14), our findings demonstrate that TLR2-induced cytokine production is not enough to ameliorate the early-stage immunosuppression induced by SARS-CoV-2 in alveolar epithelial type II cells. However, at a later timepoint a potential stimulation of TLR2 by SARS-CoV-2 can induce inflammatory and anti-viral responses (CXCL5, IFNα) and promote disease pathogenesis by enhancing the coagulation mechanism (induction of PAI-1). Several studies have found elevated levels of PAI-1 in hospitalized COVID-19 patients (4, 44, 45). Increased levels of PAI‐1 are associated with thrombosis, since inhibition of plasminogen activators results to reduced conversion of plasminogen to plasmin which degrades fibrin clots (46). PAI-1 is expressed in different cell types and its increase can be induced by proinflammatory cytokines, indicating a cross-link between inflammation and thrombosis (47, 48). Moreover, studies have shown that PAI-1 is highly induced in alveolar type II cells in idiopathic pulmonary fibrosis (IPF), regulating alveolar type II cell senescence and secretion of profibrotic mediators (49). Nevertheless, our data show a modest induction of PAI-1 expression in TLR2 activated cells, suggesting that SARS CoV2 S protein signaling may not be the primary signal inducing PAI-1. The simultaneous increase of CXCL5, responsible for neutrophil recruitment, also correlates with the development of immunothrombosis, since neutrophils are known to stabilize microthrombi via the release of neutrophil extracellular traps (NETs) (50).

To decipher the molecular pathway by which SARS-CoV-2 S protein exerts this early immunosuppressive action in alveolar epithelial cells, we treated A549 cells with SARS-CoV-2 S protein, TLR2 ligand PAM3csk4 and their combination for 15 and 30 minutes, in order to investigate their impact to specific molecular pathways, such as PI3K/AKT and MAPK/ERK. Our results demonstrated that SARS-CoV-2 S protein did not affect the MAPK/ERK pathway, but significantly induced the PI3K/AKT pathway, since the induction of AKT phosphorylation was observed upon stimulation with SARS-CoV-2 S protein, particularly when co-stimulated with PAM3csk4. To further verify that the immunosuppressive effects of SARS-CoV-2 were mediated through the activation of PI3K/AKT, we also treated A549 cells with the selective AKT 1/2/3 inhibitor MK2206 and measured the production of IL8, IL6 and TNFα after S protein stimulation compared to mock treated cells. Stimulation with PAM3csk4 was not performed at this instance, since SARS-CoV-2 S protein is the primary cause of the decreased inflammatory responses observed, while PAM3csk4 induces inflammatory responses independently through TLR2 receptor. Indeed, we observed that SARS-CoV-2 S was not able to significantly alter the production of IL8, IL6 and TNFα in MK2206-treated cells compared to mock treated cells. Our findings are in accordance with other studies indicating the possible implication of the PI3K/AKT pathway in the immunosuppressive action of the virus and other diseases. For example, IL-37, a member of the IL-1 family which is stimulated by SARS-CoV-2, has been shown to suppress IL-1β, IL-6, TNFα and CCL2 in rheumatic diseases by acting on mTOR and enhancing the AMPK activity, to maintain mitochondrial membrane potential and limit the toxic effects of ROS (51). Other studies have also shown that PI3K signaling mediates an immunosuppressive phenotype in myeloid cells to prevent excessive innate immunity in chronic infections and inflammation (52). Another possible immunosuppressive activity of SARS-CoV-2 acting through the PI3K/AKT/mTOR pathway is the inhibition of autophagy. Studies have shown that coronaviruses might inhibit the autophagic mechanism by increasing viral replication and through upregulation of AKT/mTOR, since mTORC1 is known to inhibit autophagy (53). For example, the related corona virus MERS-CoV can interfere with host cell autophagy by promoting the degradation of BECN1, after AKT1 activation (54). Hence, Akt/mTOR inhibitors could prove a valuable asset in COVID-19 management. Various studies have shown that blockade of mTOR can inhibit protein synthesis and thus reduce viral replication and inflammation (55–57). Moreover, mTOR inhibitors can limit the proliferation of memory B cells and T cell responses, preventing the production of cross‐reactive antibodies for SARS‐CoV‐2 (58, 59).

Our study has several limitations, since the work was performed in a single cell line A549 and investigated a single molecular pathway (PI3K/AKT). In addition, the implication of the PI3K/AKT pathway could be further investigated with mTOR inhibitors or other downstream AKT targets to delineate the exact mechanism by which PI3K/AKT decreases the pro-inflammatory responses. Even if our study correlates with other studies showing decreased cytokine production in SARS- CoV-2 infection as previously mentioned, there are not many reports for cytokine production at the early stages of the infection and further research should be performed to this direction, before we can reach certain conclusions. However, this study can propose the potential use of AKT/mTOR inhibitors for the regulation of inflammatory responses during SARS-CoV-2 infection.

## Data availability statement

The raw data supporting the conclusions of this article will be made available by the authors, without undue reservation.

## Author contributions

CT, AA-Q, EV, and GS designed the study, IP performed experiments, IP, EV, FA, and HA, SM-N analyzed data, IP, CT, AA-Q, and EV drafted the manuscript, IP, CT, AA-Q, EV, FA, HA, SM-N, and GS reviewed the manuscript. All authors contributed to the article and approved the submitted version.

## Funding

This work has been funded by the Hellenic Foundation for Research and Innovation grant (HFRI, General Secretariat for Research and Technology, GSRT Grant No 1010), the King Abdullah International Medical Research Center under grant number RC17/128/R, and the King Faisal Specialist Hospital and Research Center, Riyadh, Saudi Arabia.

## Conflict of interest

The authors declare that the research was conducted in the absence of any commercial or financial relationships that could be construed as a potential conflict of interest.

## Publisher’s note

All claims expressed in this article are solely those of the authors and do not necessarily represent those of their affiliated organizations, or those of the publisher, the editors and the reviewers. Any product that may be evaluated in this article, or claim that may be made by its manufacturer, is not guaranteed or endorsed by the publisher.
