# Supplementary material for: SARS-CoV-2 modulates inflammatory responses of alveolar epithelial type II cells via PI3K/AKT pathway
Source: Front Immunol. 2022 Oct 31;13:1020624. doi: 10.3389/fimmu.2022.1020624 (PMC9659903; doi:10.3389/fimmu.2022.1020624)
Supplement: Supplementary file 1 [file DataSheet_1.pdf]

Supplementary Material

A. **B.**

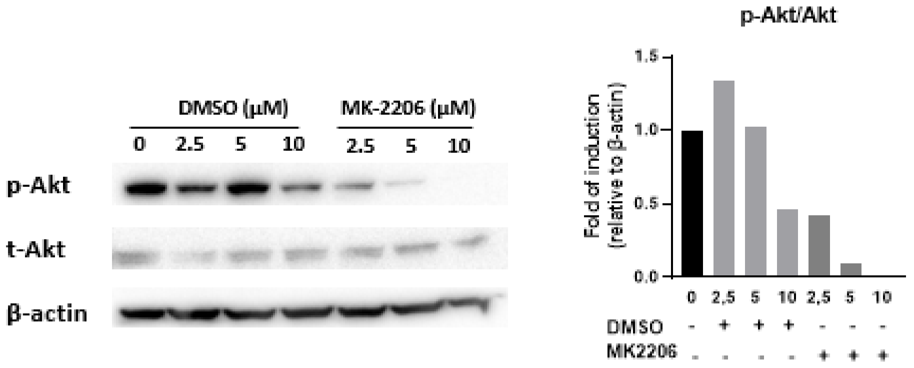

Supplementary Figure 1. Alveolar epithelial A549 cells were treated with different concentrations of the specific Akt inhibitor MK-2206 or its diluent DMSO (2.5, 5, 10 μM) for 24 h. Treatment with Akt inhibitor MK-2206 resulted in decreased Akt phosphorylation. Representative western blot of three independent experiments (A) and densitometry of the same blot (B) are depicted. Statistical testing was omitted because of the small sample size (n=1).
